# Immunoreactivity of the fully humanized therapeutic antibody PankoMab-GEX™ is an independent prognostic marker for breast cancer patients

**DOI:** 10.1186/s13046-015-0152-7

**Published:** 2015-05-19

**Authors:** Sabine Heublein, Doris Mayr, Markus Egger, Uwe Karsten, Steffen Goletz, Martin Angele, Julia Gallwas, Udo Jeschke, Nina Ditsch

**Affiliations:** Department of Gynaecology and Obstetrics, Ludwig-Maximilians-University of Munich, Marchioninistrasse 15, 81377 Munich, Germany; Department of Pathology, Ludwig-Maximilians-University of Munich, Munich, Germany; Glycotope GmbH, Berlin-Buch, Germany; Department of Surgery, Ludwig-Maximilians-University of Munich, Munich, Germany

**Keywords:** PankoMab-GEX™, Prognosis, Breast cancer, MUC1, TA-MUC1, Immunohistochemistry

## Abstract

**Background:**

Mucin-1 (MUC1, CD227), more widely known as CA15-3, is an abundantly expressed epithelial cell surface antigen and has evolved to be the most predictive serum tumour marker in breast cancer. PankoMab-GEX™, which is currently being evaluated for its therapeutic efficacy in a phase IIb clinical trial, is a glyco-optimized anti-MUC1 antibody specifically recognizing a tumour-associated MUC1 epitope (TA-MUC1). The current study aimed to analyse the immunoreactivity of PankoMabGEX™ and its correlation with established clinico-pathological variables including 10-year and overall survival in a large cohort of breast cancer patients.

**Methods:**

Breast cancer tissue sections (n = 227) underwent a standardized immunohistochemical staining protocol for TA-MUC1 by using PankoMab-GEX™ as a primary antibody. The staining was evaluated by two independent observers and quantified by applying the IR-score.

**Results:**

TA-MUC1 as detected by PankoMab-GEX™ was identified in 74.9% of breast cancer tissue sections. Patients were subdivided according to the subcellular localisation of TA-MUC1 and cases classified as mem-PankoMab-GEX™ (solely membranous) positive, cyt-PankoMab-GEX™ (solely cytoplasmic) positive, double positive or as completely negative were compared regarding their survival. Herein mem-PankoMab-GEX™-positive patients performed best, while double-negative ones presented with a significantly shortened survival. Positivity for mem-PankoMab-GEX™ as well as a double-negative immunophenotype turned out to be independent prognosticators for survival.

**Conclusions:**

This is the first study to report on PankoMab-GEX™ in a large panel of breast cancer patients. The PankoMab-GEX™ epitope TA-MUC1 could be identified in the majority of cases and was found to be an independent prognosticator depending on its subcellular localisation. Since TA-MUC1 is known to be highly immunogenic cancers staining positive for PankoMab-GEX™ might be more compromised by host anti-tumour immune defence. Further, the observations reported here might be fundamental for selecting patients to undergo PankoMab-GEX™-containing chemotherapy protocols.

**Electronic supplementary material:**

The online version of this article (doi:10.1186/s13046-015-0152-7) contains supplementary material, which is available to authorized users.

## Background

Mucin-1 (MUC1, CD227), more widely known as CA15-3, has evolved to be one of the best validated breast cancer serum tumour markers [1]. In general, MUC1 is a transmembrane cell surface glycoprotein expressed by cells of epithelial origin. Its extracellular domain is composed of a variable number of repeats with multiple glycans attached to the protein backbone. Although MUC1 is expressed on both normal and cancerous cells, there exist significant differences regarding glycosylation [2,3], expression level [4] and subcellular localisation [5] comparing normal and neoplastic cells.

PankoMab-GEX™ is a novel, meanwhile fully humanized and glyco-optimized anti-MUC1 antibody selectively recognizing a glyco-epitope termed TA-MUC1 (Tumour-Associated MUC1). TA-MUC1 has been characterized as a carbohydrate-induced conformational epitope [6,7]. Structure wise it comprises a PDTRP (Pro-Asp-Thr-Arg-Pro) motif containing a specifically glyco-modified threonine residue (T) [3,6-8]. Glycans linked to this threonine by O-glycosylation are tumour specific carbohydrate modifications themselves. Presence of this specifically glyco-modified PDTRP motif is regarded to be an absolute requirement for binding of PankoMab-GEX™ [6]. Scatchard plot analysis performed in breast cancer cell lines revealed high binding affinity (up to K_D_ = 7.1×10^−9^ M) and epitope density (up to 2.4×10^6^ per cell) of the murine IgG1, κ PankoMab antibody which is a precursor of the humanized PankoMab-GEX™ [6]. TA-MUC1 has been identified to be predominantly expressed on cancer cells while being virtually absent from the corresponding non-malignant tissue. Cancer selectivity has been demonstrated for a range of cancer entities including lung [9], ovarian [4], cervical [10] or hepatocellular carcinoma [10]. To the best of our knowledge there are only few publications having studied TA-MUC1 in breast cancer tissue and none of them included specific information on PankoMab-GEX™ in healthy breast tissue [10-12].

In addition, PankoMab-GEX™ mediates antibody-dependent cellular cytotoxicity (ADCC) [6], therefore being anticipated to potentially evolve as an excellent therapeutic antibody. Thus to further enhance its ADCC activity glyco-optimization in proprietary human glyco-engineered production cell lines (GlycoExpress™ platform) was employed. Since PankoMab-GEX™ is internalized by tumour cells in a temperature and time-dependent manner, additional efforts have been undertaken to investigate its potency in antigen-dependent toxin-mediated elimination of cancer cells [6]. Currently the unconjugated antibody is being evaluated for its therapeutic efficacy in a double-blind, randomized, placebo-controlled phase IIb clinical trial in ovarian cancer patients.

Some previous studies on the immunoreactivity of PankoMab-GEX™ have been published [4,9,10,12,13]. For instance the murine PankoMab antibody was applied in a lung cancer study [9] and in a study to discriminate sera of patients suffering from benign *versus* malignant disease of the ovary [13]. However evaluation of PankoMab-GEX™ immunoreactivity as correlated to routine clinico-pathological variables and survival in a large panel of breast cancer patients is missing so far. Hence the present study aimed to analyse PankoMab-GEX™ immunostaining with regard to the aforementioned parameters.

## Methods

### Patients

Formalin-fixed, paraffin-embedded (FFPE) breast cancer samples from 227 patients who underwent surgery due to a malignant tumour of the breast at the Department of Gynaecology and Obstetrics, Ludwig-Maximilians-University of Munich, Germany were included in this study (Table 1). Histopathological tumour subtypes were assigned according to the WHO criteria, and tumour grading was determined according to the Elston and Ellis criteria [14] by a gynecological pathologist (D.M.). Data regarding hormone receptors (ER, PR, Her2), patient age and overall survival were retrieved from patients’ charts or from the Munich Cancer Registry, respectively. None of the patients (n = 227) had a positive family history for breast cancer. Mean patient age was 58.2 ± 13.3 years. More than half of all patients were diagnosed for a breast tumour smaller than 2 cm in size (n (pT1) = 153 (68.0%), n (pT2) = 66 (29.3%), n (pT3) = 1 (0.4%), n (pT4) = 5 (2.2%)) and for cancer without lymph node metastasis (pN0: 56.7%), with a significant number of cases also displaying a DCIS/LCIS fraction within the invasive carcinomas. Mean overall survival was 12.2 years (95% CI: 11.6 - 12.8 years), mean follow-up was 9.8 years (95% CI: 9.29 - 10.4 years), and 49 deaths were documented. Further patients’ characteristics are listed in Table 1. This study has been performed and presented according to the REMARK criteria [15].

**Table 1:** **Patient characteristics**

		**n**	**%**
**Histology**			
	**NST**	131	57.7
	**non NST**	96	42.3
**Grading**			
	**G1. G2**	103	66.0
	**G3**	53	34.0
**pT**			
	**pT1**	153	68.0
	**pT2-pT4**	72	32.0
**pN**			
	**pN0**	122	56.7
	**pN1-pN3**	93	43.3
**CIS (fraction within the invasive carcinoma)**			
	**no**	107	47.1
	**yes**	120	52.9
**ER**			
	**negative**	30	14.6
	**positive**	175	85.4
**PR**			
	**negative**	62	32.6
	**positive**	128	67.4
**Her2**			
	**negative**	160	88.9
	**positive**	20	11.1
**age (years)**			
	**mean ± STDV**	58.2 ± 13.3	

### Immunohistochemistry

Tissue samples were fixed in buffered formalin solution (3.7%) immediately after resection and underwent standardized paraffin embedding. Slides were stained using PankoMab-GEX™ (final concentration: 2 μg/ml in PBS) as described before [4,11]. Human endometrium tissue served as positive control for PankoMab-GEX™ staining as described elsewhere [4], while replacement of the primary antibody with human IgG was performed as negative control.

PankoMab-GEX™ immunoreactivity was examined by two independent observers by consensus. Samples were assessed by applying an established semiquantivative immunoreactive score (IRS) [4,11,16]. The IR score quantifies immunoreactivity by multiplication of staining intensity (graded as 0 = no, 1 = weak, 2 = moderate, and 3 = strong staining) and percentage of positively stained cells (0 = no staining, 1 = ≤ 10% of the cells, 2 = 11–50% of the cells, 3 = 51–80% of the cells and 4 = ≥ 81% of the cells). A Leitz (Wetzlar, Germany) microscope was employed, and representative images were taken by a CCD colour camera (JVC, Japan). In accordance with previously published data, tissue samples with an IRS higher than 2 - regarding membranous PankoMab-GEX™ staining - were scored as positive [17,18]. Since cytoplasmic PankoMab-GEX™ staining was found to be quite low, the threshold was set at an IR score of 0 with cases scored as IRS higher than 0 counted as positive.

### Statistics

The IBM statistic package SPSS (version 22) was used to analyse the data for statistical significance. Microsoft Excel as well as Microsoft PowerPoint were employed to illustrate data. Fisher’s exact test was used to test nominal data for independence. Survival data were compared by using Kaplan-Meier graphics, and differences in patient survival times were tested for significance by calculating chi-square statistics of the log rank test. Multivariate analysis was performed using the Cox-regression model. Data were assumed to be statistically different in case of p < 0.05.

### Ethics statement

The tissue samples used were left over material after all diagnostics had been completed and were retrieved from the archive of Gynaecology and Obstetrics, Ludwig-Maximilians-University, Munich, Germany. Patient data were fully anonymised. The study was approved by the Ethics Committee of the Ludwig-Maximilians-University, and was performed according to the standards set in the declaration of Helsinki 1975.

## Results

### The PankoMab-GEX™ epitope TA-MUC1 is related to clinicopathological parameters

**Figure 1:**
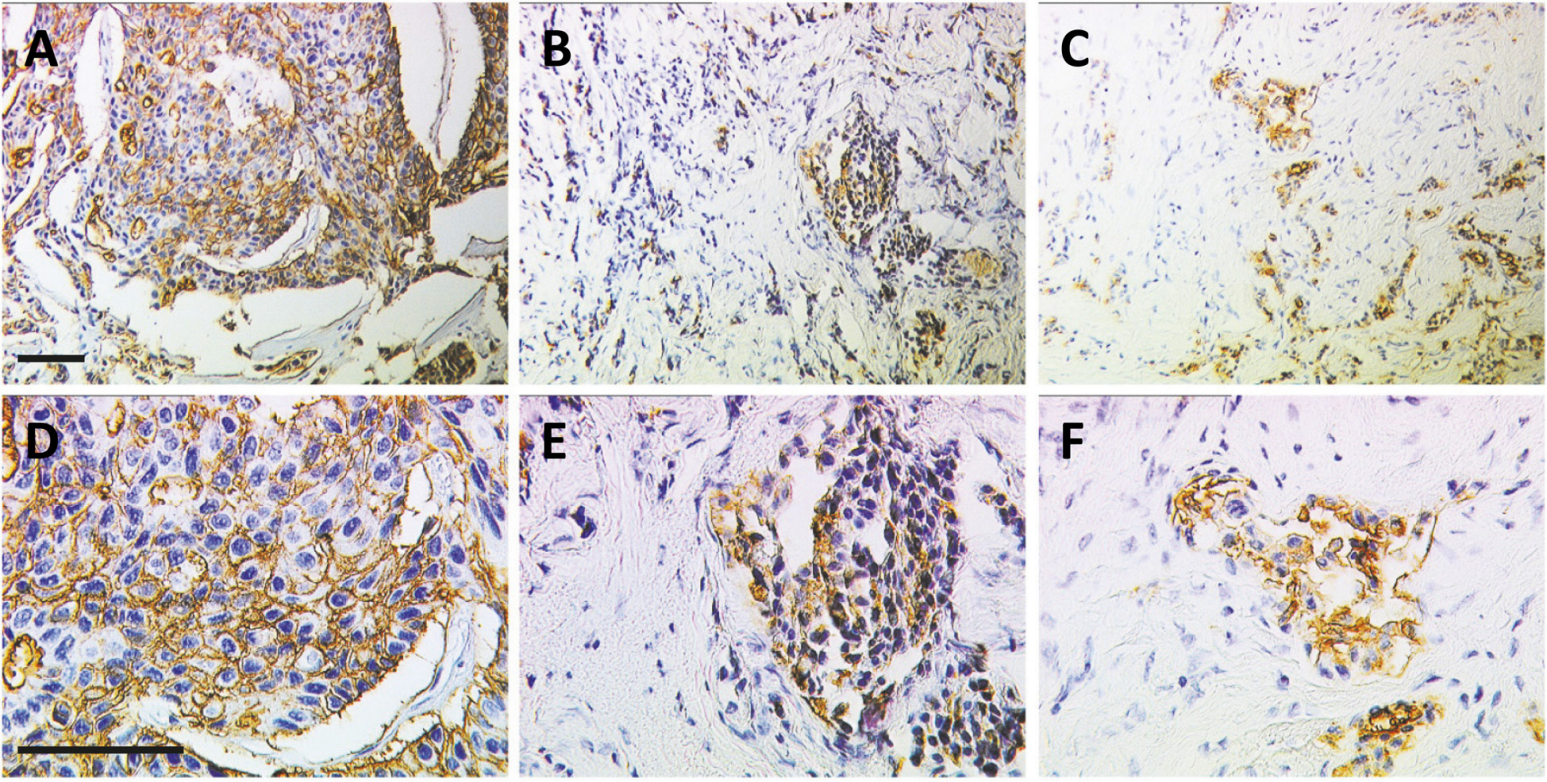
PankoMabGEX™ immunoreactivity in breast cancer tissue. Representative images of mem-PankoMab-GEX™ positive **(A, D)**, cyt-PankoMab-GEX™ positive **(B, E)** and double-positive **(C, F)** cases were distinguished. The scale bars in **A** and **D** equal 100 μm and apply to **A-C** and **D-F**, respectively.

PankoMab-GEX™ staining was restricted to tumour cells, whereas breast stroma remained negative (Figure 1). TA-MUC1 as detected by PankoMab-GEX™ was identified in 74.9% (170/227) of breast cancer tissue sections. Patients were then subdivided according to the subcellular localisation of TA-MUC1. Solely membranous (mem-) PankoMab-GEX™ positivity was observed in 40.1% (91/227) of tissue samples, while exclusively cytoplasmic (cyt-) PankoMab-GEX™ staining accounted for only 5.7% (13/227). About one third of cancers (29.1%, 66/227) showed both membranous and cytoplasmic staining (referred to as ‘double positive’) while 25.1% (57/227) of samples studied did not stain at all (referred to as ‘double negative’).

Though mem-PankoMab-GEX™ was inversely associated with tumour size (p = 0.002) and lymph node status (p = 0.001), no such correlation was observed in case of cytoplasmic staining. Neither membrane nor cytoplasmic staining was associated with histological subtype, tumour grade, or with the presence of a DCIS/LCIS component, respectively (Table 2). Further, TA-MUC1 as detected by PankoMab-GEX™ was analysed in respect to hormone receptor and Her2 positivity. Progesterone receptor (PR) positive cancers showed mem-PankoMab-GEX™ staining more often than those being PR negative (p = 0.028). An adverse association of borderline significance was seen in case of mem-PankoMab-GEX™ and Her2 positivity (p = 0.053). Cyt-PankoMab-GEX™ was not associated with either hormone receptor or Her2 positivity. Cancer tissues which were negative for TA-MUC1 at all were significantly less often found to be of NST histology (p = 0.008) and to show less ER (p = 0.008) or PR (p = 0.041). On the other hand, double positive cases revealed significantly more frequently to be of NST histology (p = 0.026), to be larger in tumour size (p = 0.041) and to be lymph node positive (p = 0.011).

**Table 2:** **Distribution of PankoMabGEX** **™** **staining patterns**

		**Mem-PankoMabGEX**					**Cyt-PankoMabGEX**					**Double negative**					**Double positive**				
		**Other**		**Mem only**			**Other**		**Cyt only**			**No**		**Yes**			**No**		**Yes**		
		**n**	**(%)**	**n**	**(%)**	**p**	**n**	**(%)**	**n**	**(%)**	**p**	**n**	**(%)**	**n**	**(%)**	**p**	**n**	**(%)**	**n**	**(%)**	**p**
**Histology**																					
	**NST**	79	(34.8)	52	(22.9)	ns	122	(53.7)	9	(4.0)	ns	107	(47.1)	24	(10.6)	0.008	85	(37.4)	46	(20.3)	0.026
	**non NST**	57	(25.1)	39	(17.2)		92	(40.5)	4	(1.8)		63	(27.8)	33	(14.5)		76	(33.5)	20	(8.8)	
**Grading**																					
	**G1. G2**	59	(37.8)	44	(28.2)	ns	98	(62.8)	5	(3.2)	ns	79	(50.6)	24	(15.4)	ns	73	(46.8)	30	(19.2)	ns
	**G3**	34	(21.8	19	(12.2)		50	(32.1)	3	(1.9)		38	(24.4)	15	(9.6)		37	(23.7)	16	(10.3)	
**pT**																					
	**pT1**	82	(36.4)	71	(31.6)	0.002	146	(64.9)	7	(3.1)	ns	116	(51.6)	37	(16.4)	ns	115	(51.1)	38	(16.9)	0.041
	**pT2-pT4**	54	(24.0)	18	(8.0)		66	(29.3)	6	(2.7)		52	(23.1)	20	(8.9)		44	(19.6)	28	(12.4)	
**pN**																					
	**pN0**	62	(28.8)	60	(27.9)	0.001	114	(53.0)	8	(3.7)	ns	96	(44.7)	26	(12.1)	ns	94	(43.7)	28	(13.0)	0.011
	**pN1-pN3**	68	(31.6)	25	(11.6)		88	(40.9)	5	(2.3)		67	(31.2)	26	(12.1)		56	(26.0)	37	(17.2)	
**CIS**																					
	**no**	65	(28.6)	42	(18.5)	ns	102	(44.9)	5	(2.2)	ns	78	(34.4)	29	(12.8)	ns	76	(33.5)	31	(13.7)	ns
	**yes**	71	(31.3)	49	(21.6)		112	(49.3)	8	(3.5)		92	(40.5)	28	(12.3)		85	(37.4)	35	(15.4)	
**ER**																					
	**negative**	22	(10.7)	8	(3.9)	ns	29	(14.1)	1	(0.5)	ns	17	(8.3)	13	(6.3)	0.008	22	(10.7)	8	(3.9)	ns
	**positive**	99	(48.3)	76	(37.1)		165	(80.5)	10	(4.9)		141	(68.8)	34	(16.6)		120	(58.5)	55	(26.8)	
**PR**																					
	**negative**	43	(22.6)	19	(10.0)	0.028	59	(31.1)	3	(1.6)	ns	42	(22.1)	20	(10.5)	0.041	42	(22.1)	20	(10.5)	ns
	**positive**	66	(34.7)	62	(32.6)		121	(63.7)	7	(3.7)		105	(55.3)	23	(12.1)		92	(48.4)	36	(18.9)	
**Her2**																					
	**negative**	90	(50.0)	70	(38.9)	ns	150	(83.3)	10	(5.6)	ns	119	(66.1)	41	(22.8)	ns	121	(67.2)	39	(21.7)	ns
	**positive**	16	(8.9)	4	(2.2)		18	(10.0)	2	(1.1)		14	(7.8)	6	(3.3)		12	(6.7)	8	(4.4)	
**age**																					
	**≤55 y**	57	(25.2)	33	(14.6)	ns	85	(37.6)	5	(2.2)	ns	59	(26.1)	31	(13.7)	0.012	69	(30.5)	21	(9.3)	ns
	**>55 y**	79	(35.0)	57	(25.2)		128	(56.6)	8	(3.5)		110	(48.7)	26	(11.5)		91	(40.3)	45	(19.9)	

### PankoMab-GEX™ is an independent prognostic marker in breast cancer patients

**Figure 2:**
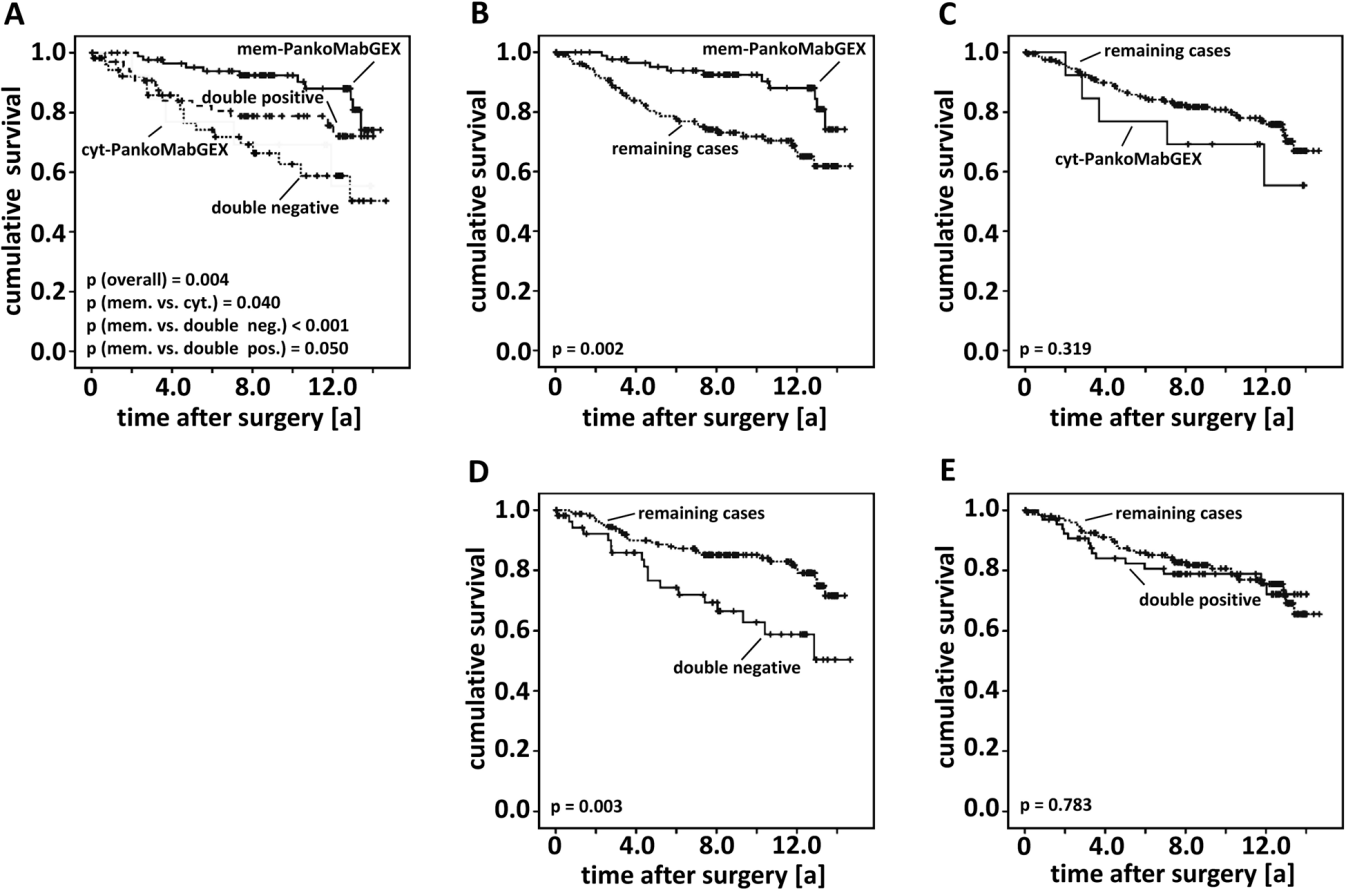
PankoMabGEX™ predicts overall survival in breast cancer. Univariate analysis revealed significant differences regarding overall survival of the four subgroups (mem-PankoMab-GEX™ positive, cyt-PankoMab-GEX™ positive, double negative and double positive) studied **(A)**. mem-PankoMab-GEX™ positivity turned out to be related to more favourable **(B)** survival, while a double negative immunophenotype was associated with worse prognosis **(D)**. Neither cyt-PankoMab-GEX™ positivity **(C)** nor a double positive immunophenotype **(E)** was predictive for overall survival. The term “remaining cases” refers to the three immunophenotypes different from the one indicated in the respective graph **(B-E)**.

The several groups of cases were analysed regarding their ten year and overall survival. Patients classified as positive for mem-PankoMab-GEX™ presented with a significantly more favourable survival than those diagnosed for other staining locations (Figure 2A; Additional file 1: Figure S1A). Similarly, mem-PankoMab-GEX™ positive cases indicated better survival compared the group of remaining (i.e. cyt-PankoMab-GEX™ positive, double negative and double positive) cases (overall survival: p = 0.002, Figure 2B; 10-year survival: p < 0.001, Additional file 1: Figure S1B). In cases where TA-MUC1 was not expressed at all (double-negative) the overall survival rates (p = 0.003, Figure 2D) as well as the 10-year (p = 0.002, Additional file 1: Figure S1D) were found to be significantly reduced compared to the remaining cases. Finally, there was no significant difference observed when survival rates of cyt-PankoMab-GEX™ positive or double-positive patients were contrasted to the remaining cases, respectively (Figure 2C, E; Additional file 1: Figure S1C, E).

Multivariate Cox-Regression analysis was performed in order to test whether mem-PankoMab-GEX™ staining or the total absence of PankoMab-GEX™ staining (‘double-negative group’) might be prognosticators of 10-year or overall survival. Besides Her2-positivity, the diagnosis of being double-negative for the PankoMab-GEX™ turned out to be an independent negative prognosticator for 10-year survival (Table 3), though both failed to be of prognostic significance regarding multivariate analysis of overall survival rates. More importantly, the mem-PankoMab-GEX™ immunophenotype proved to be an independent positive predictor for 10-year (p = 0.022) as well as overall (p = 0.042) survival. ER positivity as well as a positive lymph node status was a further independent predictor in the current analysis (Table 4).

**Table 3:** **Absence of PankoMabGEX** **™** **staining is an independent prognosticator for reduced 10-year survival**

	**10-year survival**				
			**95% CI**		
**Covariate**	**Coefficient (b**_**i**_ **)**	**[HR Exp (b**_**i**_ **)]**	**Lower**	**Upper**	**P-value**
Histology (NST vs. other)	−0.94	0.39	0.09	1.71	ns
Grading (G1. G2 vs. G3)	−0.39	0.68	0.19	2.47	ns
pT (pT1 vs. pT2-pT4)	−0.03	0.97	0.34	2.80	ns
pN (pN0 vs. pN1-pN3)	0.68	1.98	0.64	6.11	ns
CIS (fraction within the invasive carcinoma) (no vs. yes)	0.05	1.05	0.39	2.85	ns
ER (neg. vs. pos.)	−1.20	0.30	0.06	1.42	ns
PR (neg. vs. pos.)	0.26	1.30	0.37	4.62	ns
Her2 (neg. vs. pos.)	1.45	4.25	1.07	16.94	0.040
age (≤55y vs. > 55 y.)	0.74	2.09	0.69	6.31	ns
PankoMabGEX™ double negative (no vs. yes)	1.30	3.67	1.12	12.08	0.032

**Table 4:** **mem-PankoMabGEX** **™** **staining is an independent prognosticator for advanced 10-year and overall survival**

	**10-year survival**					**Overall survival**				
			**95% CI**					**95% CI**		
**Covariate**	**Coefficient (b**_**i**_ **)**	**[HR Exp (b**_**i**_ **)]**	**Lower**	**Upper**	**P-value**	**Coefficient (b**_**i**_ **)**	**[HR Exp (b**_**i**_ **)]**	**Lower**	**Upper**	**P-value**
Histology (NST vs. other)	−0.49	0.61	0.16	2.38	ns	−0.31	0.73	0.23	2.34	ns
Grading (G1. G2 vs. G3)	−0.51	0.60	0.18	1.97	ns	−0.59	0.55	0.19	1.63	ns
pT (pT1 vs. pT2-pT4)	−0.17	0.84	0.30	2.36	ns	−0.06	0.94	0.39	2.26	ns
pN (pN0 vs. pN1-pN3)	0.61	1.84	0.63	5.40	ns	1.06	2.89	1.11	7.53	.030
CIS (fraction within the invasive carcinoma) (no vs. yes)	0.08	1.08	0.40	2.97	ns	0.28	1.32	0.57	3.06	ns
ER (neg. vs. pos.)	−1.53	0.22	0.05	0.94	.041	−1.66	0.19	0.05	0.80	.023
PR (neg. vs. pos.)	0.10	1.10	0.28	4.28	ns	0.37	1.45	0.43	4.91	ns
Her2 (neg. vs. pos.)	0.92	2.52	0.56	11.35	ns	0.67	1.95	0.46	8.20	ns
age (≤55y vs. > 55 y.)	0.75	2.11	0.71	6.30	ns	0.28	1.32	0.55	3.18	ns
mem-PankoMabGEX™ (neg. vs. pos.)	−2.42	0.09	0.01	0.70	.022	−1.21	0.30	0.09	0.96	.042

## Discussion

Since subcellular localisation of certain MUC1 isoforms has been associated with tumour cell aggressiveness and cancer prognosis [19-21], this relationship was also studied using the antibody PankoMab-GEX™, which detects a tumour-associated epitope of MUC1 (TA-MUC1) [6]. Our study revealed that exclusive membrane-associated TA-MUC1 staining (measured as mem-PankoMab-GEX™) was inversely correlated with tumour size (pT stage) and lymph node metastasis (pN stage). However, since more than half of all samples were classified as pT1 the association of mem-PankoMab-GEX™ and pT-stage might partly be biased by sampling. In line with Vegt et al. [22] the absence of both membranous and cytoplasmic TA-MUC1 (‘double negative’ immunophenotype) was often found in tumours characterized as hormone receptor-negative. MUC1 has been shown to interact with hormone receptors e.g. by stabilizing ER and by stimulating ER-mediated transcription [23], hereby indicating a functional interaction of MUC1 and hormone receptor signalling in breast cancer. Nevertheless, concordant blockage of MUC1 and ER has failed to be effective in a randomized phase II trial performed with locally advanced, metastasized breast cancer [24]. The current study detected some relations of TA-MUC1 staining and hormone receptors, too. Presence of mem-PankoMab-GEX™ was associated with PR positivity. Further, staining of mem-PankoMab-GEX™ showed a trend of being negatively associated with Her2 positivity. With Her2 being known to indicate poor prognosis in breast cancer [25-27] and with mem-PankoMab-GEX™ staining predicting just the opposite an adverse relationship of both markers was expected.

More importantly, the current study reports a positive correlation of membrane-associated TA-MUC1 with prolonged survival. On the opposite, cases in which staining with PankoMab-GEX™ was also seen in the cytoplasm, or was absent at all, presented with shorter survival periods. Studies with other anti-MUC1 antibodies obtained similar results regarding apical expression of MUC1 and its correlation with better overall survival [20,22] and low metastatic potential [19]. Cytosolic localisation of MUC1, on the other hand, has been found to be elevated in breast cancer [28] and to protect pancreatic cancer cells from undergoing apoptosis [29].

A similar association of PankoMab-GEX™ expression and prognosis has previously been reported in unifocal breast cancer [12] and in lymph node-positive lung cancer [9]. Two effects may contribute to the improved survival of patients with exclusively membranous expression of TA-MUC1. First, membranous localisation of MUC1 - as opposed to cytoplasmic localisation or total loss of expression - is an indicator of a more differentiated tumour. Second, modified MUC1, as long as it is exposed at the cell membrane, is immunogenic. MUC1 is known to induce autoantibody formation in cancer patients [30-32]. The TA-MUC1 epitope recognized by PankoMab-GEX™ is an immunodominant antigenic region of MUC1 representing a mixed peptide-carbohydrate conformational epitope [6] that involves either of two carbohydrate structures termed TF (Thomsen-Friedenreich antigen) or Tn (TF precursor) [33]. We thus hypothesize that breast cancer cells displaying mem-PankoMab-GEX™ positivity are predisposed to be recognized by endogenous or newly induced anti-MUC1 auto-antibodies potentially acting via ADCC mechanisms, as demonstrated for PankoMab-GEX™ [6]. Finally, our observations reported here may also be fundamental for selecting patients to undergo PankoMab-GEX™-containing chemotherapy protocols.

## Conclusions

In conclusion, this study highlighted the TA-MUC1 epitope as detected by PankoMab-GEX™ to be widely expressed in a large body of breast cancer tissue samples, and - in case of its membrane-restricted expression - to be an independent predictor for better survival. Hence our results demonstrate that PankoMab-GEX™, which has been developed primarily as a therapeutic antibody, may also serve as a diagnostic tool.

## Additional file

Additional file 1: Figure S1. PankoMabGEX™ predicts 10-year survival in breast cancer. Univariate analysis revealed significant differences regarding 10-year survival of the four subgroups (mem-PankoMab-GEX™ positive, cyt-PankoMab-GEX™ positive, double negative and double positive) studied (A). mem-PankoMab-GEX™ positivity turned out to be related to more favourable (B) survival, while a double negative immunophenotype was associated with worse prognosis (D). Neither cyt-PankoMab-GEX™ positivity (C) nor a double positive immunophenotype (E) was predictive for 10-year survival. The term “remaining cases” refers to the three immunophenotypes different from the one indicated in the respective graph (B-E).

## Abbreviations

TA-MUC1: Tumour-Associated Mucin-1
mem-PankoMab-GEX™: Solely membranous staining
cyt-PankoMab-GEX™: Solely cytoplasmic staining
PDTRP: Pro-Asp-Thr-Arg-Pro (amino acid motif)
ADCC: Antibody-dependent cellular cytotoxicity
FFPE: Formalin-fixed paraffin-embedded
REMARK: Reporting Recommendations for Tumor Marker Prognostic Studies
PBS: Phosphate-buffered saline
IRS: Immunoreactive score
NST: No-special type
CIS: Carcinoma in situ component within / alongside the invasive cancer

## References

[1] Harris L, Fritsche H, Mennel R, Norton L, Ravdin P, Taube S (2007). American society of clinical oncology 2007 update of recommendations for the use of tumor markers in breast cancer. J Clin Oncol.

[2] Cao Y, Blohm D, Ghadimi BM, Stosiek P, Xing PX, Karsten U (1997). Mucins (MUC1 and MUC3) of gastrointestinal and breast epithelia reveal different and heterogeneous tumor-associated aberrations in glycosylation. J Histochem Cytochem.

[3] Karsten U, Serttas N, Paulsen H, Danielczyk A, Goletz S (2004). Binding patterns of DTR-specific antibodies reveal a glycosylation-conditioned tumor-specific epitope of the epithelial mucin (MUC1). Glycobiology.

[4] Dian D, Lenhard M, Mayr D, Heublein S, Karsten U, Goletz S (2013). Staining of MUC1 in ovarian cancer tissues with PankoMab-GEX detecting the tumour-associated epitope, TA-MUC1, as compared to antibodies HMFG-1 and 115D8. Histol Histopathol.

[5] Fukushima M, Higuchi K, Shimojo H, Uehara T, Ota H (2012). Distinct cytoplasmic expression of KL-6 mucin in chromophobe renal cell carcinoma: a comparative immunohistochemical study with other renal epithelial cell tumors. Acta Histochemica et Cytochemica.

[6] Danielczyk A, Stahn R, Faulstich D, Loffler A, Marten A, Karsten U (2006). PankoMab: a potent new generation anti-tumour MUC1 antibody. Cancer Immunol Immunother.

[7] Karsten U, von Mensdorff-Pouilly S, Goletz S (2005). What makes MUC1 a tumor antigen?. Tumour Biol.

[8] Karsten U, Diotel C, Klich G, Paulsen H, Goletz S, Muller S (1998). Enhanced binding of antibodies to the DTR motif of MUC1 tandem repeat peptide is mediated by site-specific glycosylation. Cancer Res.

[9] Kuemmel A, Single K, Bittinger F, Faldum A, Schmidt LH, Sebastian M (2009). TA-MUC1 epitope in non-small cell lung cancer. Lung Cancer.

[10] Fan XN, Karsten U, Goletz S, Cao Y (2010). Reactivity of a humanized antibody (hPankoMab) towards a tumor-related MUC1 epitope (TA-MUC1) with various human carcinomas. Pathol Res Pract.

[11] Dian D, Janni W, Kuhn C, Mayr D, Karsten U, Mylonas I (2009). Evaluation of a novel anti-mucin 1 (MUC1) antibody (PankoMab) as a potential diagnostic tool in human ductal breast cancer; comparison with two established antibodies. Onkologie.

[12] Weissenbacher T, Hirte E, Kuhn C, Janni W, Mayr D, Karsten U (2013). Multicentric and multifocal versus unifocal breast cancer: differences in the expression of E-cadherin suggest differences in tumor biology. BMC Cancer.

[13] Jeschke U, Wiest I, Schumacher AL, Kupka M, Rack B, Stahn R (2012). Determination of MUC1 in sera of ovarian cancer patients and in sera of patients with benign changes of the ovaries with CA15-3, CA27.29, and PankoMab. Anticancer Res.

[14] Elston EW, Ellis IO (1993). Method for grading breast cancer. J Clin Pathol.

[15] McShane LM, Altman DG, Sauerbrei W, Taube SE, Gion M, Clark GM (2005). Reporting recommendations for tumor marker prognostic studies (REMARK). J Natl Cancer Inst.

[16] Scholz C, Toth B, Barthell E, Mylonas I, Weissenbacher T, Friese K (2009). Immunohistochemical expression of glycodelin in breast cancer correlates with estrogen-receptor alpha and progesterone-receptor A positivity. Histol Histopathol.

[17] Lenhard M, Tereza L, Heublein S, Ditsch N, Himsl I, Mayr D (2012). Steroid hormone receptor expression in ovarian cancer: progesterone receptor B as prognostic marker for patient survival. BMC Cancer.

[18] Lenhard M, Tsvilina A, Schumacher L, Kupka M, Ditsch N, Mayr D (2012). Human chorionic gonadotropin and its relation to grade, stage and patient survival in ovarian cancer. BMC Cancer.

[19] Guo Q, Tang W, Inagaki Y, Kokudo N, Sugawara Y, Karako H (2007). Subcellular localization of KL-6 mucin in colorectal carcinoma cell lines: association with metastatic potential and cell morphology. Oncol Rep.

[20] Guo Q, Tang W, Inagaki Y, Midorikawa Y, Kokudo N, Sugawara Y (2006). Clinical significance of subcellular localization of KL-6 mucin in primary colorectal adenocarcinoma and metastatic tissues. World J Gastroenterol.

[21] Luna-More S, Rius F, Weil B, Jimenez A, Bautista MD, Perez-Mellado A (2001). EMA: a differentiation antigen related to node metastatic capacity of breast carcinomas. Pathol Res Pract.

[22] van der Vegt B, de Roos MA, Peterse JL, Patriarca C, Hilkens J, de Bock GH (2007). The expression pattern of MUC1 (EMA) is related to tumour characteristics and clinical outcome of invasive ductal breast carcinoma. Histopathology.

[23] Wei X, Xu H, Kufe D (2006). MUC1 oncoprotein stabilizes and activates estrogen receptor alpha. Mol Cell.

[24] Ibrahim NK, Yariz KO, Bondarenko I, Manikhas A, Semiglazov V, Alyasova A (2011). Randomized phase II trial of letrozole plus anti-MUC1 antibody AS1402 in hormone receptor-positive locally advanced or metastatic breast cancer. Clin Cancer Res.

[25] Krishnamurti U, Silverman JF (2014). HER2 in breast cancer: a review and update. Adv Anat Pathol.

[26] Nair R, Roden DL, Teo WS, McFarland A, Junankar S, Ye S (2014). c-Myc and Her2 cooperate to drive a stem-like phenotype with poor prognosis in breast cancer. Oncogene.

[27] Mu Z, Klinowska T, Dong X, Foster E, Womack C, Fernandez SV (2014). AZD8931, an equipotent, reversible inhibitor of signaling by epidermal growth factor receptor (EGFR), HER2, and HER3: preclinical activity in HER2 non-amplified inflammatory breast cancer models. J Exp Clin Cancer Res.

[28] de Oliveira JT, Pinho SS, de Matos AJ, Hespanhol V, Reis CA, Gartner F (2009). MUC1 expression in canine malignant mammary tumours and relationship to clinicopathological features. Vet J.

[29] Banerjee S, Mujumdar N, Dudeja V, Mackenzie T, Krosch TK, Sangwan V (2012). MUC1c regulates cell survival in pancreatic cancer by preventing lysosomal permeabilization. PLoS One.

[30] Burford B, Gentry-Maharaj A, Graham R, Allen D, Pedersen JW, Nudelman AS (2013). Autoantibodies to MUC1 glycopeptides cannot be used as a screening assay for early detection of breast, ovarian, lung or pancreatic cancer. Br J Cancer.

[31] Von Mensdorff-Pouilly S, Moreno M, Verheijen RH (2011). Natural and induced humoral responses to MUC1. Cancers (Basel).

[32] Isla Larrain M, Demichelis S, Crespo M, Lacunza E, Barbera A, Creton A (2009). Breast cancer humoral immune response: involvement of Lewis y through the detection of circulating immune complexes and association with Mucin 1 (MUC1). J Exp Clin Cancer Res.

[33] Goletz S, Cao Y, Danielczyk A, Ravn P, Schoeber U, Karsten U (2003). Thomsen-Friedenreich antigen: the “hidden” tumor antigen. Adv Exp Med Biol.

