# Supplementary figures and images for: Immunoreactivity of the fully humanized therapeutic antibody PankoMab-GEX™ is an independent prognostic marker for breast cancer patients
Source: J Exp Clin Cancer Res. 2015 May 19;34(1):50. doi: 10.1186/s13046-015-0152-7 (PMC4447018; doi:10.1186/s13046-015-0152-7)

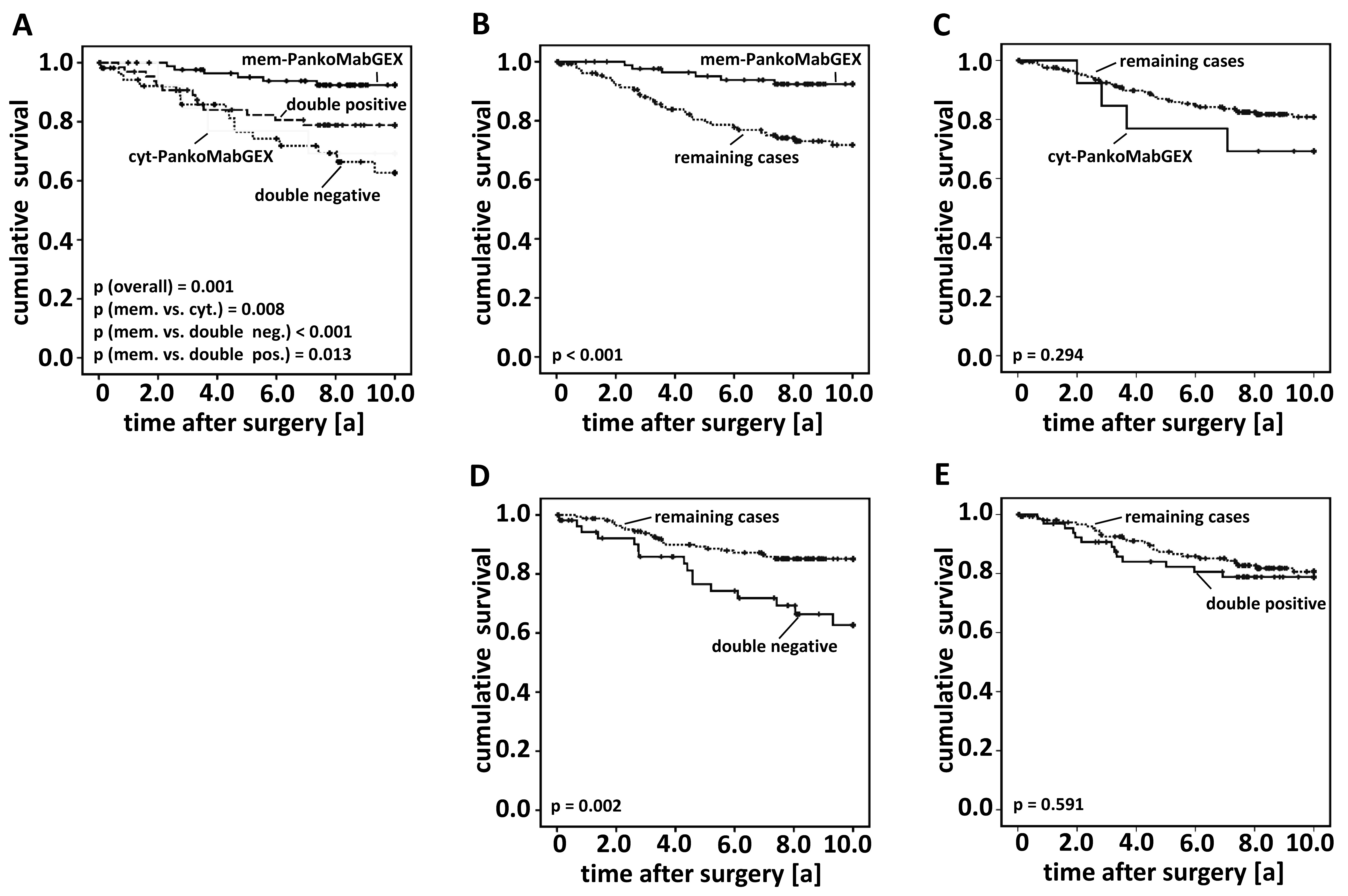

Supplement: Additional file 1: Figure S1. — PankoMabGEX™ predicts 10-year survival in breast cancer. Univariate analysis revealed significant differences regarding 10-year survival of the four subgroups (mem-PankoMab-GEX™ positive, cyt-PankoMab-GEX™ positive, double negative and double positive) studied (A). mem-PankoMab-GEX™ positivity turned out to be related to more favourable (B) survival, while a double negative immunophenotype was associated with worse prognosis (D). Neither cyt-PankoMab-GEX™ positivity (C) nor a double positive immunophenotype (E) was predictive for 10-year survival. The term “remaining cases” refers to the three immunophenotypes different from the one indicated in the respective graph (B-E). [file 13046_2015_152_MOESM1_ESM.jpg]
